# Prevalence and Possible Predictors of Gambling Disorder in a Sample of Students in the Healthcare Professions

**DOI:** 10.3390/ijerph20010452

**Published:** 2022-12-27

**Authors:** Francesca Scandroglio, Giulia Ferrazzi, Alessia Giacobazzi, Vera Vinci, Mattia Marchi, Gian Maria Galeazzi, Alessandro Musetti, Luca Pingani

**Affiliations:** 1 Department of Biomedical, Metabolic and Neural Sciences, Università degli Studi di Modena e Reggio Emilia, 41125 Modena, Italy; 2 Dipartimento ad attività integrata di Salute Mentale e Dipendenze Patologiche, Azienda USL-IRCCS di Reggio Emilia, 42122 Reggio Emilia, Italy; 3 Department of Humanities, Social Sciences and Cultural Industries, University of Parma, 43121 Parma, Italy; 4 Direzione delle Professioni Sanitarie, Azienda USL-IRCCS di Reggio Emilia, 42122 Reggio Emilia, Italy

**Keywords:** gambling, pathological gambling, health professions students, students, SOGS

## Abstract

The Italian version of the South Oaks Gambling Screen questionnaire (SOGS) and a socio-demographic questionnaire were administered to a sample of 275 healthcare professions students aged 19 to 58 years (mean age = 22.17; females = 81.1%) to address the research objectives: to examine the prevalence and correlates of problem gambling in a population of university healthcare professions students in Italy. Among the sample, 8.7% (*n* = 24) of participants showed problem gambling and 1.5% (*n* = 4) pathologic gambling. Lottery and scratch cards were the most frequent type of gambling in the sample, followed by cards and bingo. Compared to females, males tend to be more involved in problem gambling and pathological gambling. Males tend to be more involved than females in different types of gambling (such as cards, sports bets, gambling at the casino). Pathological gambling is positively associated with gender, being students lagging behind the regular schedule of exams and parents’ level of education. These findings have important implications in terms of prevention and intervention on gambling and pathological gambling. Universities should make available educational programs and counselling services to address this issue.

## 1. Introduction

Gambling can be defined as “an activity that involves placing something of value at risk in the hopes of gaining something of greater value” [1] and can be practiced in different ways: playing cards, betting on sports, lotteries, scratch cards and slot machines for example.

The evolution of gambling environments has created new gambling modalities, such as online gambling, which has significantly expanded over the past few years [1,2]. Moreover, the online gambling market has also grown due to the COVID-19 pandemic [3].

Gambling legislation is extremely heterogeneous: in Italy, gambling is considered illegal when it is practiced in a public place or in a private club. Instead, it is legal when it is authorised by the State: it can be managed by the Customs and State Monopolies Agency or by private operators authorised by the State (for example, casinos) [4]. In the United States, instead, it is legal under the US federal law, although significant restrictions pertaining to interstate and online gambling are present. Each state can also decide to regulate or prohibit gambling within its borders [5].

In recent years, the definition of gambling disorder has changed significantly. In the Diagnostic and Statistical Manual of Mental Disorders- Fifth Edition (DSM-5), pathological gambling was moved from the “Impulse Control Disorders Not Elsewhere Classified” category to “Substance-Related and Addictive Disorders” [6,7]. Moreover, the American Psychiatry Association [8] in the DSM-5 has reclassified and renamed pathological gambling as “gambling disorder”: a persistent and recurrent problematic behaviour which leads to significant impairment or distress for a period of at least 12 months. Criteria include persistent thoughts and preoccupations about gambling, gambling with greater amounts of money to reach the same level of tolerance and to achieve the desired excitement, several unsuccessful efforts to reduce or to stop gambling, restlessness and/or irritability and/or withdrawal connected to these efforts and the interference of gambling in the most important areas of life functioning (e.g., relationships, job, education). People who suffer from gambling disorder can gamble when feeling distressed (e.g., anxious, depressed), can lie to hide the extent of their involvement in gambling, may rely on other’s money to relieve bad financial situations caused by gambling and can try to regain recent gambling-related losses (“chasing” losses).

It is important to note that the literature on gambling presents discrepancies due to different terms used to describe this phenomenon: the term problem gambling is generally used to indicate a precursor of pathological gambling and in the literature both these problems are considered as a part of disordered gambling [9].

Gambling disorder has demonstrated to have a high comorbidity with other mental health conditions [3,10,11]. Indeed, pathological gambling can be associated with previous mental disorders (e.g., anxiety, mood and substance abuse disorders) and can also predict the onset of generalised anxiety disorder, post-traumatic stress disorder and substance dependence [12]. In addition to the severity of this problem, among those with gambling problems only a few (7–29%) seek help and treatment [3,13].

### 1.1. Prevalence of Gambling Disorder in the General Population

A recent meta-analysis (2016) [14] showed that the prevalence of gambling has various rates worldwide (0.12–5.8%) and in Europe (0.12–3.4%). According to the authors, it is challenging to compare studies due to different methodological procedures, instruments, cut-offs and time frames. According to the classification of the DSM-IV ([8]), the prevalence of lifetime pathological gambling in large-scale epidemiological studies lies in a range between the 0.4% and the 0.6% of the general population in the United States [1,12,15]. In the United Kingdom, estimates range from 0.6% to 0.9% of the population [16]. In Germany, the prevalence is estimated to be between 0.2% and 0.6% and according to this review most pathological gamblers are men (79–80%) and more than 90% of patients suffer from other mental health issues [17]. In Australia, the prevalence rate of problem gambling ranges between 0.5% and 2% of the adult population, with another 2% being moderately at risk [18]. According to a comparative study [19], 4% and 1.8% of the general population of Hong Kong can be classified respectively as problem gamblers and pathological gamblers.

Data available from the Italian context, covering the period from 2013 to 2018, estimate problem gamblers to be between 1.3% and 3.8% of the Italian population and the rate of pathological gamblers is estimated to vary from 0.5% and 2.2% of the population [20,21]. In addition, according to the Italian Population Survey on Alcohol and other Drugs conducted in 2017 by the National Council of Research, 42.8% of the Italian population aged between 15 and 64 years had gambled at least once in the past 12 months [22]. As described by the Italian Gambling Observatory, the identikit of the Italian “problem gambler” can be described as follows: male, adult, attends technical or professional institutes with insufficient academic performance, resident in the South, with family or friends who are also gamblers [23]. It is worth to note that higher estimates of pathological gambling have been reported in specific clinical populations [1]. For example, higher rates of pathological gambling were found among individuals with psychiatric disorders [24] and the disorder often co-occurs with substance use disorder, mood disorders, impulse-control disorders and medical/neurological conditions [1,25,26,27,28].

When examining epidemiological studies, we should consider that research conducted with screening instruments have frequently shown a higher prevalence of this disorder, compared with studies which used the DSM criteria [29]. Moreover, an important change in the DSM-5 classification consisted in the reduction of the threshold of the inclusion criteria to 4 out of 9 and this modification seems to have increased underestimation of the prevalence of the disorder [8,29].

Several specific risk factors for problem gambling have been identified. Several studies confirmed that among the socio-demographic characteristics, male gender [19,30,31,32] and a low level of education [33,34] are those most recognised and studied as well as substance use problems, high level of anxiety and a poor quality of life [1]. College students are one of the populations most at risk for pathological gambling [35]: college years are often associated with behaviours such as drinking and drug use; exam pressure can be a source of stress that is difficult to manage; accumulated tuition debt can seem insurmountable. A situation therefore of potential frustration that can open the door to pathological gambling [36].

### 1.2. Gambling among College and University Students

An empirical synthesis of pathological gambling research was done in 1999 in United States and Canada by Shaffer and colleagues [15]. The Authors calculated an overall estimate rate of 5.05% (95% CI of 3.55, 6.56%) in college students in 14 studies conducted between 1987 and 1997, considering the most used assessing instrument, which was the South Oaks Gambling Screen questionnaire (SOGS) [9,15]. Another meta-analysis published by Blinn-Pike [37] considered 15 studies between 1999 and 2005 which used the SOGS to assess the presence of disordered gambling. This study calculated an estimated proportion of gambling disorders among college students at 7.9% (95% CI of 5.37, 10.41%). A more recent meta-analysis considered 18 studies conducted between 2005 and 2013 and estimated the proportion of pathological gamblers in college students at 10.23% (95% CI of 7.17, 13.29%); this meta-analysis also included studies conducted outside North America [38]. The latest meta-analysis available was published in 2018 [9]: the estimated percentage of college students that could be classified as pathological gamblers is 6.13% (95% CI of 5.19, 7.07%) with an additional 10.23% classified as problem gamblers (95% CI of 7.79, 12.68%). Therefore, problem and pathological gambling affect millions of students worldwide and these issues need to be addressed both through close collaboration between research institution and health agencies: this articulated synergy will permit to optimise therapeutic interventions, in order not to waste public finances and to identify early interventions for states at risk [9].

### 1.3. Students in the Healthcare Professions and Gambling

Kavan et al. [39], in 2012, conducted a study on medical students at Creighton University (US) (*n* = 418), using the SOGS. 61% of participants reported gambling at least once in the previous year. Among these, 17.6% were at risk of developing problem gambling, according to the SOGS scoring. Overall, 11.7% of the participants and 19.2% of gamblers scored one point or more on the SOGS. Regarding the type of gambling, slot machines were used in 56.5% of the cases and playing cards with friends in 55.7%, followed by lotto (45.9%), casino card games (44.9%) and sporting events (36.1%). According to this study, gambling was found to be a common activity among medical students: medical students involved in gambling were more likely to be male, to have consumed marijuana over the past 12 months and to be younger than the non-gambler peers [39].

Another study, published in 2009 and always conducted at Creighton University (US), described gambling among dentistry students using the SOGS [40]. 61.3% of respondents (*n* = 186) reported having gambled at least once in the last 12 months. An Italian study on 1.083 nursing students, data collected from June to October 2015, reported that, according to the SOGS scores, 83.3% of the sample showed problem gambling and 2.7% pathological gambling [41]. According to this study, students with problem gambling were more likely to be male and to suffer from anxiety [41]. A more recent cross-sectional study conducted in Italy in 2019 on nursing students found that on a sample of 413 subjects, 9.4% showed problem gambling, according to the “Canadian Problem Gambling Index” [42]. This study also showed that the probability to be classified as “Player at risk/Moderate gambling problems/Serious game problems” was significantly associated with male gender, not living with family members, low perceived health status and risky consumption of alcohol [42].

University life necessarily includes stressful elements (for example the coordination between study and work, the payment of tuition fees and home rent, having an inactive and limited social life) which can lead students, directly or indirectly, to seek a solution to their problems in gambling: the last Young Gamers and Gamblers Education Trust (YGAM) survey reported that 47% of students have gambled in the last 12 months and among these, 16% have been identified as moderate risk or problem gamblers [43].

The purposes of this study are three: To describe problem gambling rates in the student population of health professions degree programs; examine gender differences regarding gambling-related behaviour; examine socio-demographic characteristics associated with problem gambling.

## 2. Materials and Methods

### 2.1. Participants and Procedure

This study was conducted in the Academic Year 2020/2021 and was approved by the Ethics Committee Ethics Committee “Area Vasta Emilia Nord” (protocol numbers 2020/0089330 and 0021122/20).

An email requesting participation in an online survey was sent to a convenience sample of 1.383 healthcare professions students to the University of Modena and Reggio Emilia, with the aim of understanding the extent and the characteristics of gambling among participants. Among the 1383 students who received the request for participating in our study, 275 (19.88%) accepted to be involved.

The inclusion criteria were to be enrolled in a bachelor’s degree of healthcare professions of the University of Modena and Reggio Emilia (https://international.unimore.it/ (accessed on 19 December 2022)), a university in the North of Italy (Biomedical Laboratory Techniques, Cardiocirculatory and Cardiovascular Perfusion Techniques, Dental Hygiene, Dietetics, Imaging and Radiotherapy Techniques, Midwifery, Nursing, Occupational Therapy, Physiotherapy, Psychiatric Rehabilitation Technique and Speech and Language Therapy). The research project was illustrated to each participant who was also asked for informed consent, which was necessary to be involved in the study. Once consent was given, each participant was asked to fill in a socio-demographic form and the Italian version of the SOGS.

### 2.2. Measures

#### 2.2.1. Sociodemographic Characteristics

Participants were asked about socio-demographic data, such as age, gender, university course attended, being students lagging behind the regular schedule of exams and/or working students, geographical area of residence, family composition, parents’ level of education. In addition, students answered a question about the presence of mental health issues in their family. Finally, the questionnaire contained two questions on the reasons that led a person to gamble, and on gambling awareness events they had previously participated in.

#### 2.2.2. South Oaks Gambling Screen

The Italian version of the SOGS [44] was used to assess problem gambling as it represents one of the most used tools at an international level for the evaluation of the presence of pathological gambling. It consists of 16 questions, of these, only 12 are used to calculate the total score: the non-scoring items identify type of gambling, amount of money gambled in a day and relatives/friends with gambling problems. The first question aims to identify the frequency with which respondents play the different types of gambling; all other questions use the “yes/no” answer mode.

The questionnaire was created in English language [45] validated in several languages and specific subpopulations (e.g., adolescent) demonstrating excellent psychometric qualities [46,47,48].

The final score is calculated as follows: one point if “yes” was answered to question 4 for the option “most of the time I lose” or “every time I lose”; one point if “yes” was answered to question 5 for the option “yes, less than half of the times I’ve lost” or “ yes, most of the time”; one point if “yes” was answered to question 6 for the option “yes, in the past, but not now” or “ yes”; one point for each single “yes” to questions 7 to 11; one point for each single “yes” to questions 13 to 16i (except 16j and 16k which are not calculated).

The total score can therefore vary from 0 to 20. The cut off in the Italian version is described as follows: no problem (Total score < 3); problem, at-risk gambler (3 ≤ Total score < 5); pathological gambler (Total score ≥ 5).

The Cronbach’s Alpha of the questionnaire obtained in our study is equal to 0.88.

### 2.3. Data Analysis

First, we performed descriptive analysis (absolute and percentage frequencies for quantitative variables; mean and standard deviation for qualitative variables) to examine the rate of problem and pathological gambling in the sample. We used the Chi-Square test to compare the categorical variables. Then, linear regression was used to investigate possible predictors of problem and pathological gambling.

## 3. Results

Age of participants ranged from 19 to 58 years (mean = 22.17; SD = ±3.79). Females were 81.09% (*n* = 223) of the sample. Most participants were from the north of Italy (*n* = 243; 88.36%;) and lived with their parents (*n* = 218; 79.27%). A total of 68 participants (24.73%) had family members with mental illness. About 59.27% (*n* = 163) of participants’ parents had a high school diploma.

Among the participants, 82.18% (*n* = 226) were full-time students and 26.55% (*n* = 73) had previously participated in at least one awareness event (such as congress and/or seminars) on pathological gambling. About 72.36% (*n* = 199) of the students believed the motive for gambling to be “pure fun, entertainment, thrill, emotion”, 22.18% (*n* = 61) “to increase income”, 5.45% (N = 15) believed it could be a way to “test own abilities”. Socio-demographic characteristics of the sample are shown in Table 1.

Results on gambling-related variables are shown in Table 2. The types of gambling most practiced (once or more times a week) were playing cards (*n* = 4; 1.45%) and sports betting (*n* = 7; 2.55%). Playing cards (*n* = 52; 18.91%), scratch cards (*n* = 122; 44.36%) and bingo (*n* = 39; 14.18%) were the most practiced with less frequency than once a week. More than half of the students (*n* = 155; 56.36%) wagered a maximum of EUR 10 in one single day. Only 1.82% of the sample (*n* = 5) had at least one parent with gambling problems. About 19 students (6.91%) have sometimes returned to gambling to recover lost money while only 2 (0.72%) acknowledged that they have or have had problems with pathological gambling. 9 study participants (3.27%) reported being criticised for their gambling while 10 students (3.64%) wagered more than they wanted or felt guilty. If borrowed money was used to gamble or to pay off debts, 1.82% (*n* = 5) used house money, 1.09% (*n* = 3) used their partner’s money and 0.73% (*n* = 2) used other relatives’ money or credit card. Only one student (0.36%) had money in stock, bonds, or other securities.

According to the SOGS cut-off, most of the respondents (*n* = 247; 89.82%) did not show a problem with gambling while the 8.73% (*n* = 24) showed some problems with gambling. Four students (1.45%) met the cut-off for pathological gambling.

Table 3 describes the absolute and percentage frequencies of responses to the SOGS by gender. Among the different types of gambling, only for the game of dice and bingo there was no statistically significant difference: males were more involved in card games (Χ^2^ = 17.98; df = 2; *p* < 0.001) and sports betting (Χ^2^ = 89.90; df = 2; *p* < 0.001), whereas females in the use of scratch cards (Χ^2^ = 13.30; df = 2; *p* = 0.001). The percentage of males and females who lost at most in a day EUR 10 or less at gambling is almost equal (56.86% vs. 56.50%) and no statistically significant difference was identified between the two subpopulations regarding the presence of one or both parents suffering from pathological gambling (Χ^2^ = 0.006; df = 1; *p* = 0.65). Returning to gambling to recover the money previously lost is practiced more by the male subpopulation than by the female one: 13.73% returned to play some time (versus 5.38%) and 3.92% returned to play most of the times after a loss (versus 0.90%) (Χ2 = 19.27; df = 4; *p* = 0.001). Statistically significant differences were found regarding the perception of having spent more than intended (M: 66.66%–F: 43.17%) (Χ^2^ = 17.21; df = 2; *p* < 0.001) and being criticised for gambling (M: 13.73%–F: 0.90%) (Χ^2^ = 28.71; df = 2; *p* < 0.001). Having hidden from partner, family members or important people the evidence of gambling (betting slips, lottery tickets, gambling money, etc.,) was more present in the male subpopulation (21.74% versus 1.18%) (Χ^2^ = 14.59; df = 1; *p* < 0.001). Having less time for work (or school) because of gambling was more present in the male subpopulation (3.92%) than in the female one (N = 0) (Χ^2^ = 8.81; df = 1; *p* = 0.03).

Finally, in the male subpopulation we found a greater number of individuals at risk (*n* = 14; 27.45%) and suffering from pathological gambling (N = 3; 5.88%) than in the female one (at risk: *n* = 10; 4.48%-pathological gambling: *n* = 1; 0.45%) (Χ^2^ = 37.12; df = 2; *p* < 0.001).

All the conditions for running the multiple linear regression were met: minimum sample size required (N = 105), the presence of correlation of the predictors with the criterion variable, the normal distribution and the non-presence of multicollinearity (Table 4). Hypothetical predictors of pathological gambling (Table 4) were male gender (β = −0.29; *p* < 0.001), being students lagging behind the regular schedule to complete the course (β = 4.18; *p* < 0.001) and having parents with low level of education (β = −2.80; *p* = 0.005).

## 4. Discussion

This study aimed at investigating problem and pathological gambling rates and associated socio-demographic characteristics in a sample of health professions university students.

Descriptive analysis showed that only 4 students (1.43%) were identified as probable pathological gamblers, while 24 (8.73%) as problem gamblers and 247 (89.92%) did not show any problem gambling. Comparing these results with the previous literature we can observe interesting differences. Kavan and colleagues in 2012 [39] found that 17.6% of medical student (Creighton University-US) involved in the study were at risk of developing pathological gambling. Two years before, Elsasser and colleagues [2], in the Creighton University (US), described similar results: 25.9% of participants (Pharmacy Students) were identified at risk of developing a problem gambling. Instead, the results discussed in 2018 by Cicolini and colleagues [41] concerning an Italian Nursing course, were much closer to ours: a prevalence of 2.7% of problem gambling on a nurse students’ population while the same phenomenon in our study was about 1.45%. However, Cicolini and colleagues [41] involved 1.083 students, while our sample size was limited and could have influenced the real number of the investigated phenomenon (fear of being stigmatised may have a major impact on the decision to participate).

The second goal of this study was to define gender differences related to the gambling phenomenon. The previous literature on the general population and the student population of the health professions describes how the prevalence of pathological gambling is higher in the male population [2,38,39,49,50,51]. In our study, the prevalence in the male subpopulation for at-risk gambler was about 27.45% (*n* = 14) and probable pathological gamblers are 5.88% (N = 3). At the same time female at-risk are 4.48% (*n* = 10) and only one is a probable pathological gambler (0.45%). Our study confirmed these data.

Furthermore, it is interesting to highlight some differences in the approach to games between genres. Males are more involved in card games and sports betting while females are major users of scratch cards. In the literature only few studies have investigated the difference between gambling modality and how the types of game impacts the development of gambling-related pathology [52]. Betting on different sports was the most popular option also in a sample of Spanish students [36]. Interestingly, researcher suggests that there is a need for the development of transversal strategies to raise awareness of the potential dangers of gambling in students: the normalisation of sports betting within the cultural context can represent a critical element for vulnerable subjects already experiencing mental distress [53].

We can also identify different behaviours between the two subpopulations regarding gaming-related spending. Our research shows a similar percentage of males and females that affirm to spend EUR 10 or less for the gaming activity. Overall, the literature from UK (2012) and Finland (2015) shows that men tend to spend more money on gambling than women [54,55]. This has been made clear for both younger and older adults [33,56]. Hiding betting slips, lottery tickets, gambling money or other signs of gambling from a spouse, children or other important people is more present in the male subpopulation (21.74% versus 1.18%) in a significant statistical difference. A similar result has been previously described in 2014 in UK [55]: males experience increased feelings of guilt and shame and this can be hypothesised that it may also lead them to hide the evidence of their action.

Another interesting and significant difference between male and female subpopulations is represented by the criticism received for gambling. More males claim to have received disapproval about their gambling while only few females underwent the same situation. We can speculate that being more criticised is associated with higher levels of money spent and higher frequency of gambling.

The present study has allowed to denote some behavioural and emotional differences related to gambling in the male and female subgroups: we believe it is important to proceed with further studies on these subpopulations in order to better define precise prevention and intervention projects for the pathological gambling.

The result obtained from the multiple linear regression analysis showed that pathological gambling was positively associated with the male gender [30,32,57]. To the best of our knowledge, no other studies have identified being students lagging behind the regular schedule of exams as a possible predictor. It is also difficult to hypothesise whether this risk factor may or may not be considered as a consequence of practicing pathological gambling. The last one association identified is a low parental education level that is confirmed by the previous literature in the general population [58]. Family characteristics seem to play a key role in explaining the presence of gambling problems in adolescent population [59]. Moreover, lower family life satisfaction has also been related to a feeling of rejection, in this way engaging in gambling is motivated by a desire to modulate specific psychological needs [60]. In this way, a systematic review published in 2017, supports the fact that a warm family environment offers the opportunity to receive emotional support to reduce the risk of problem gambling [61].

It is necessary to highlight the limitations of the study. First, we used a convenience sample which cannot be generalised with the entire student population of the Health Professions Degree Courses of the Faculty of Medicine and Surgery or with the general population of the same age group. The response rate obtained is low because only 275 students (19.88%) decided to participate in the stud. Furthermore, we can hypothesise that among the students who have decided not to participate there are some sufferings from pathological gambling: the fear of stigmatisation or even of facing the topic may have caused their non-compliance. Second, we have decided to undertake this study in a stressful period. The COVID-19 pandemic has changed the lives of all people in the world and has influenced styles and habits, we can assume that consequently even the gambling habits may have changed as well. For instance, in 2021 in Italy evidence state that the modality and frequency of games have changed in the population from a game played in real place to an online game [62]. This new condition could have significantly affected the actual frequency of game which shows differences from what we have expected based on previous research available. Third, females were 81.09% (*n* = 223) of the sample and therefore the results may be conditioned by this distribution (which in any case represents that of the entire student population of the Faculty of Medicine and Surgery). Finally, it is necessary to consider results obtained by the SOGS with caution: the main criticism against the SOGS argues that it seems to overestimate the prevalence of pathological gambling [63,64].

## 5. Conclusions

The phenomenon of gambling is increasingly available and new gambling products are constantly being developed. Despite the overall number of students identified in several epidemiological studies, gambling does not receive all the necessary attention from the institutions. The results of this study, despite the limitations shown, can therefore represent a contribution to the definition of objectives, strategies and methods for the development of gambling prevention plans in the university environment. As stated earlier, our sample does not allow us to draw firm conclusions, but it is our idea that spending time on education on this topic can have a positive influence in generating reflections on the topic of gambling. After all, the level of education is identified as a valuable factor accompanying problem gambling [31,48].

## Figures and Tables

**Table 1:** Socio-demographic characteristics of the sample.

	Mean (SD)	Minimum		Maximum
*Age*	22.17 (±3.79)	19		58
	**N**		**%**	
*Sex*				
Male	51		18.55	
Female	223		81.09	
Prefer not to answer	1		0.36	
*Being students lagging behind the regular schedule of exams*				
Yes	16		5.82%	
No	259		94.18%	
*Place of residence*				
North of Italy	243		88.36%	
Central Italy	24		8.73%	
South of Italy	8		2.91%	
*Housing condition*				
By him/herself	11		4%	
With parents	218		79.27%	
Sharing an apartment with other students	31		11.27%	
Living with partner	12		4.36%	
University residence	3		1.09%	
*Highest educational qualification held by parents*				
Primary school diploma	3		1.09	
Middle school diploma	40		14.55	
High school diploma	163		59.27	
Bachelor’s degree	55		20	
Master’s degree	14		5.09	
*Working student*				
Yes	49		17.82%	
No	226		82.18%	
*Family members with experience of mental illness*				
Yes	68		24.73%	
No	207		75.27%	
*Why does a person gamble?*				
Pure fun, recreation, thrill, emotion	199		72.36%	
To supplement income	61		22.18%	
That’s one way to test abilities.	15		5.45%	
*Have you attended outreach events (seminars, conferences, etc.) on gambling?*				
Yes	73		26.55%	
No	202		73.45%	

**Table 2:** Answers to the SOGS questionnaire (from question 1 to 6).

	**Not at All (N; %)**			**Less than Once a Week (N; %)**		**Once a Week or More (N; %)**		
Q1. Indicate which of the following types of gambling you have done in your lifetime.								
*Played cards for money*	219 (79.64%)			52 (18.91%)		4 (1.45%)		
*Bet on horses, dogs or other animals*	269 (97.82%)			6 (2.18%)		0		
*Bet on sports*	246 (89.45%)			22 (8%)		7 (2.55%)		
*Played dice games for money*	274 (99.64%)			1 (0.36%)		0		
*Went to casino (legal or otherwise)*	269 (97.82%)			5 (1.82%)		1 (0.36%)		
*Played the numbers or bet on lotteries* *(also consider scratch cards)*	150 (54.55%)			122 (44.36%)		3 (1.09%)		
*Played bingo*	235 (85.45%)			39 (14.18%)		1 (0.36%)		
*Played the stock and/or commodities market*	269 (97.82%)			3 (1.09%)		3 (1.09%)		
*Played slot machines, poker machines or* *other gambling machines*	263 (95.64%)			9 (3.27%)		3 (1.09%)		
*Bowled, shot pool, played golf or played* *some other game of skill for money*	270 (98.18%)			5 (1.82%)		0		
Q2. What is the largest amount of money you have ever gambled with any one day?	**Never have gambled (N; %)**	**EUR 10 or less** **(N; %)**		**More than EUR 10 up to EUR 100** **(N; %)**	**More than EUR 100 up to EUR 1.000** **(N; %)**	**More than EUR 1.000 up to EUR 10.000 (N; %)**		**More than EUR 10.000 (N; %)**
	96 (34.91%)	155(56.36%)		18(6.55%)	3 (1.09%)	3 (1.09%)		0
Q3. Do (did) your parents have a gambling problem?	**Both my father and mother gamble (or gambled) too much (N; %)**			**My father or my mother gambles (or gambled) too much (N; %)**		**Neither gambles (or gambled)** **too much (N; %)**		
	0			5 (1.82%)		270 (98.18%)		
Q4. When you gamble, how often do you go back another day to win back money you lost?	**Never (or never gamble)** **(N; %)**		**Some of the time (less than half the time) I lost (N; %)**		**Most of the time I lost (N; %)**		**Every time I lost (N; %)**	
	251 (91.27%)		19 (6.91%)		4 (1.45%)		1 (0.36%)	
Q5. Have you ever claimed to be winning money gambling but weren’t really? In fact, you lost?	**Never (or never gamble)** **(N; %)**			**Yes, less than half the time I lost (N; %)**		**Yes, most of the times (N; %)**		
	273 (99.27%)			2 (0.73%)		0		
Q6. Do you feel you have ever had a problem with gambling?	**No (N; %)**			**Yes, in the past, but not now (N; %)**		**Yes (N; %)**		
	273 (99.27%)			1 (0.36%)		1 (0.36%)		
	**No (or never gamble)**				**Yes**			
Q7. Did you ever gamble more than you intended?	265 (96.36%)				10 (3.64%)			
Q8. Have people criticised your gambling?	266 (96.73%)				9 (3.27%)			
Q9. Have you ever felt guilty about the way you gamble or what happens when you gamble?	265 (96.36%)				10 (3.64%)			
Q10. Have you ever felt like you would like to stop gambling but didn’t think you could?	270 (98.18%)				5 (1.82%)			
Q11. Have you ever hidden betting slips, lottery tickets, gambling money, or other signs of gambling from your spouse, children, or other important people in your life?	269 (97.82%)				6 (2.18%)			
Q12. Have you ever argued with people you like over how you handle money?	216 (78.55%)				59 (21.45%)			
Q13. (If you answered “yes” to question 12): Have money arguments ever centred on your gambling?	273 (99.27%)				2 (0.73%)			
Q14. Have you ever borrowed from someone and not paid them back as a result of your gambling?	272 (98.91%)				3 (1.09%)			
Q15. Have you ever lost time from work (or school) due to gambling?	273 (99.27%)				2 (0.73%)			
16. If you borrowed money to gamble or to pay gambling debts, where did you borrow from?								
*From household money*	270 (98.18%)				5 (1.82%)			
*From your spouse*	272 (98.91%)				3 (1.09%)			
*From other relatives or in-laws*	273 (99.27%)				2 (0.73%)			
*From banks, loan companies or credit unions*	275 (100%)				0			
*From credit cards*	273 (99.27%)				2 (0.73%)			
*From loan sharks (Shylocks)*	275 (100%)				0			
*Your cashed in stocks, bonds or other securities*	274 (99.64%)				1 (0.36%)			
*You sold personal or family property*	275 (100%)				0			
*You borrowed on your checking account* *(passed bad checks)*	275 (100%)				0			
*You have (had) a credit line with a bookie*	275 (100%)				0			
*You have (had) a credit line with a casino*	275 (100%)				0			
	**No problem** **(Total score at SOGS < 2)**			**Problematic, at-risk gambler** **(2 ≤ Total score at SOGS < 5)**		**Probable pathological gamblers** **(Total score at SOGS ≥ 5)**		
Presence of pathological gambling	247 (89.82%)			24 (8.73%)		4 (1.45%)		

**Table 3:** Differences in responses to SOGS between the male and female subpopulations.

			Male	Female	Chi Square Test
*Q1. Indicate which of the following types of gambling you have done in your lifetime*	Played cards for money	Not at all	31 (60.78%)	187 (83.86%)	Χ^2^ = 17.98; df = 2; *p* < 0.001
		Less than once a week	17 (33.33%)	35 (15.70%)	
		Once a week or more	3 (5.88%)	1 (0.45%)	
	Bet on horses, dogs, or other animals	Not at all	47 (92.16%)	221 (99.10%)	Χ^2^ = 9.35; df = 2; *p* = 0.01
		Less than once a week	4 (7.84%)	2 (0.90%)	
	Bet on sports	Not at all	27 (52.94%)	218 (97.76%)	Χ^2^ = 89.90; df = 2; *p* < 0.001
		Less than once a week	17 (33.33%)	5 (2.24%)	
		Once a week or more	7 (13.73%)	0	
	Played dice games for money	Not at all	51 (100%)	222 (99.55%)	Χ^2^ = 0.23; df = 2; *p* = 0.81
		Less than once a week	0	1 (0.45%)	
	Went to casino (legal or otherwise)	Not at all	47 (92.16%)	221 (99.10%)	Χ^2^ = 10.23; df = 2; *p* = 0.006
		Less than once a week	3 (5.88%)	2 (0.90%)	
		Once a week or more	1 (1.96%)	0	
	Played the numbers or bet on lotteries (also consider scratch cards)	Not at all	27 (52.94%)	122 (54.71%)	Χ^2^ = 13.30; df = 2; *p* = 0.001
		Less than once a week	21 (41,18%)	101 (45.29%)	
		Once a week or more	3 (5.88%)	0	
	Played bingo	Not at all	42 (82.35%)	192 (86.10%)	Χ^2^ = 4.53; df = 2; *p* = 0.10
		Less than once a week	8 (15.69%)	31 (13.90%)	
		Once a week or more	1 (1.96%)	0	
	Played the stock and/or commodities market	Not at all	45 (88.24%)	223 (100%)	Χ^2^ = 26.82; df = 2; *p* < 0.001
		Less than once a week	3 (5.88%)	0	
		Once a week or more	3 (5.88%)	0	
	Played slot machines, poker machines or other gambling machines	Not at all	45 (88.24%)	217 (97.31%)	Χ^2^ = 14.76; df = 2; *p* = 0.001
		Less than once a week	3 (5.88%)	6 (2.69%)	
		Once a week or more	3 (5.88%)	0	
	Bowled, shot pool, played golf or played some other game of skill for money	Not at all	48 (94.12%)	221 (99.10%)	Χ^2^ = 5.76; df = 2; *p* = 0.04
		Less than once a week	3 (5.88%)	2 (0.90%)	
*Q2. What is the largest amount of money you have ever gambled with any one day?*	Never have gambled		11 (21.57%)	84 (37.67%)	Χ^2^ = 17.41; df = 5; *p* = 0.004
	EUR 10 or less		29 (56.86%)	126 (56.50%)	
	More than EUR 10 up to EUR 100		8 (15.69%)	10 (4.48%)	
	More than EUR 100 up to EUR 1.000		1 (1.96%)	2 (0.90%)	
	More than EUR 1.000 up to EUR 10.000		2 (3.92%)	1 (0.45%)	
	More than EUR 10.000		0	0	
*Q3. Do (did) your parents have a gambling problem?*	Neither gamble (or gambled) too much		50 (98.04%)	219 (98.21%)	Χ^2^ = 0.006; df = 1; *p* = 0.65
	My father or my mother gambles (or gambled) too much		1 (1.96%)	4 (1.79%)	
*Q4. When you gamble, how often do you go back another day to win back money you lost?*	Never (or never gamble)		41 (80.39%)	209 (93.72%)	Χ^2^ = 19.27; df = 4; *p* = 0.001
	Some of the time (less than half the time) I lost		7 (13.73%)	12 (5.38%)	
	Most of the time I lost		2 (3.92%)	2 (0.90%)	
	Every time I lost		1 (1.96%)	0	
*Q5. Have you ever claimed to be winning money gambling but weren’t really? In fact, you lost?*	Never (or never gamble)		50 (98.04%)	222 (99.55%)	Χ^2^ = 10.32; df = 2; *p* = 0.006
	Yes, less than half the time I lost		1 (1.96%)	1 (0.45%)	
	Yes, most of the times		0	0	
*Q6. Do you feel you have ever had a problem with gambling?*	No		49 (96.08%)	223 (100%)	Χ^2^ = 8.81; df = 2; *p* = 0.012
	Yes, in the past, but not now		1 (1.96%)	0	
	Yes		1 (1.96%)	0	
*Q7. Did you ever gamble more than you intended?*	Never (or never gamble)		17 (33.33%)	126 (56.50%)	Χ^2^ = 17.21; df = 2; *p* < 0.001
	Yes, in the past, but not now		28 (54.90%)	93 (41.70%)	
	Yes		6 (11.76%)	4 (1.47%)	
*Q8. Have people criticised your gambling?*	Never gamble		17 (33.33%)	136 (60.99%)	Χ^2^ = 28.71; df = 2; *p* < 0.001
	No		27 (52.94%)	85 (38.12%)	
	Yes		7 (13.73%)	2 (0.90%)	
*Q9. Have you ever felt guilty about the way you gamble or what happens when you gamble? °*	No		27 (81.82%)	80 (95.24%)	Χ^2^ = 5.46; df = 1; *p* = 0.02
	Yes		6 (18.18%)	4 (4.76%)	
*Q10. Have you ever felt like you would like to stop gambling but didn’t think you could? °*	No		30 (90.91%)	83 (97.65%)	Χ^2^ = 2.66; df = 1; *p* = 0.10
	Yes		3 (9.09%)	2 (2.35%)	
*Q11. Have you ever hidden betting slips, lottery tickets, gambling money, or other signs of gambling from your spouse, children, or other important people in your life? °*	No		18 (78.26%)	84 (98.82%)	Χ^2^ = 14.59; df = 1; *p* < 0.001
	Yes		5 (21.74%)	1 (1.18%)	
*Q12. Have you ever argued with people you like over how you handle money?*	No		37 (72.55%)	178 (79.82%)	Χ^2^ = 1.30; df = 1; *p* = 0.17
	Yes		14 (27.45%)	45 (20.18%)	
*Q13. (If you answered “yes” to question 12): Have money arguments ever centred on your gambling?*	No		12 (85.71%)	45 (100%)	Χ^2^ = 3.01; df = 1; *p* = 0.09 *
	Yes		2 (14.29%)	0	
*Q14. Have you ever borrowed from someone and not paid them back as a result of your gambling?*	No		49 (96.08%)	222 (99.55%)	Χ^2^ = 4.62; df = 1; *p* = 0.03
	Yes		2 (3.92%)	1 (0.45%)	
*Q15. Have you ever lost time from work (or school) due to gambling?*	No		49 (96.08%)	223 (100%)	Χ^2^ = 8.81; df = 1; *p* = 0.03
	Yes		2 (3.92%)	0	
*Q16. If you borrowed money to gamble or to pay gambling debts, where did you borrow from?* *°*	From household money	No	9 (75%)	26 (92.86%)	Χ^2^ = 1.09; df = 1; *p* = 0.30 *
		Yes	3 (25%)	2 (7.14%)	
	From your spouse	No	10 (83.33%)	27 (96.43%)	Χ^2^ = 0.62; df = 1; *p* = 0.43 *
		Yes	2 (16.67%)	1 (3.57%)	
	From other relatives or in-laws	No	11 (91.67%)	27 (96.43%)	Χ^2^ = 0.03; df = 1; *p* = 0.87 *
		Yes	1 (8.33%)	1 (3.57%)	
	From banks, loan companies or credit unions	No	12 (100%)	28 (100%)	Not calculable*
		Yes	0	0	
	From credit cards	No	10 (83.33%)	28 (100%)	Χ^2^ = 2.03; df = 1; *p* = 0.15 *
		Yes	2 (16.67%)	0	
	From loan sharks (Shylocks)	No	12 (100%)	28 (100%)	Not calculable*
		Yes	0	0	
	Your cashed in stocks, bonds or other securities	No	11 (91.67%)	27 (96.43%)	Χ^2^ = 0.03; df = 1; *p* = 0.87 *
		Yes	1 (8.33%)	1 (3.57%)	
	You sold personal or family property	No	12 (100%)	28 (100%)	Not calculable *
		Yes	0	0	
	You borrowed on your checking account (passed bad checks)	No	12 (100%)	28 (100%)	Not calculable *
		Yes	0	0	
	You have (had) a credit line with a bookie	No	12 (100%)	28 (100%)	Not calculable *
		Yes	0	0	
	You have (had) a credit line with a casino	No	12 (100%)	28 (100%)	Not calculable *
		Yes	0	0	
*Presence of pathological gambling*	No problem (total score at SOGS < 2)		34 (66.67%)	212 (95.07%)	Χ^2^ = 37.12; df = 2; *p* < 0.001
	Problematic, at-risk gambler (2 ≤ Total score at SOGS < 5)		14 (27.45%)	10 (4.48%)	
	Probable pathological gamblers (Total score at SOGS ≥ 5)		3 (5.88%)	1 (0.45%)	

* Yates’s chi-squared test; ° Only students who have gambled/gamble were considered.

**Table 4:** Multiple linear regression for the definition of possible predictors of pathological gambling.

	Unstandardised Coefficients		Standardised Coefficients	t	*p*	95% Confidence Interval		Collinearity Statistics	
	*B*	*ES*	*Beta*			*Lower Bound*	*Upper Bound*	*Tolerance*	*VIF*
Sex	−0.87	0.02	−0.29	−5.04	<0.001	−1.21	−0.53	0.97	1.04
Being students lagging behind the regular schedule of exams	1.22	0.08	0.24	4.18	<0.001	0.64	1.79	0.93	1.08
Highest educational qualification held by parents	−0.25	0.29	−0.16	−2.80	0.005	−0.42	−0.07	0.98	1.02
Working student	0.03	0.09	0.01	0.18	0.86	−0.32	0.39	0.90	1.12

## Data Availability

All data used for this study are available upon request addressed to the corresponding author.
